# Complete Genome Sequence of the WHO International Standard for HIV-2 RNA Determined by Deep Sequencing

**DOI:** 10.1128/genomeA.01626-15

**Published:** 2016-02-04

**Authors:** Claire Ham, Clare Morris, Neil Berry

**Affiliations:** Division of Virology, National Institute for Biological Standards and Control, South Mimms, United Kingdom

## Abstract

The World Health Organization (WHO) International Standard for HIV-2 RNA nucleic acid assays was characterized by complete genome deep sequencing. The entire coding sequence and flanking long terminal repeats (LTRs), including minority species, were assigned subtype A. This information will aid design, development, and evaluation of HIV-2 RNA amplification assays.

## GENOME ANNOUNCEMENT

Human immunodeficiency virus type 2 (HIV-2) represents the second human lentivirus causally linked to severe immunodeficiency. The screening of blood/blood products and tissue preparations for HIV-2 RNA concentrations is an essential part of ensuring blood safety and clinical investigations. A World Health Organization (WHO) International Standard (IS) for HIV-2 RNA, prepared for genome amplification techniques, has been in use since 2009 (1). The genetic composition of the HIV-2 IS has not been described; hence we report the complete genome sequence using deep sequencing, analogous to that reported for the HIV-1 IS (2).

The HIV-2 IS was prepared from CAM-2 virus stock held at NIBSC, an HIV-2 isolate originating from Guinea-Bissau in 1987 (3), derived from viral supernatant previously described (1). Viral RNA was extracted using QiaAMP viral RNA minikits (Qiagen) and reverse transcription (RT) and amplification reactions performed with a SuperScript III one-step RT-PCR system with platinum *Taq* HiFi (Invitrogen) using primer pairs designed to generate six overlapping amplicons. Primers were 5′LTRF CTTTGGGTGGCTATGGAAGC, 5′LTRR AGGTGGTGCTGTTGGTCTAC; 1F AAGATTGTGGGAGATGGGCG, 1R AGTTCATAGCCCATCCAGCG; 2F ACCCCAGATGAGAAGTTCCAAA, 2R CCAAGAGCAGTTAGCAAGCG; 3F AGACCCCAGAGAGACCGTA, 3R TGATGTAGGTGCGAAGCCAA; 4F ATAAAAGGCCCAGGCAAGCA, 4R AGCTCCTCCCATATCCCTCC; and 3′ LTRF CACCCAGCACAAACAAGCAG, 3′ LTRR CAGTGAAGACAGGAGAGGCA. Bar-coded libraries of amplicons were made using Nextera XT library preparation kit (Illumina), and equimolar amounts of each sample sequenced in 250 bp paired-end MiSeq sequencing reactions. After removal of primer and adaptor sequences, *de novo* assembly was performed on quality trimmed and paired reads using Geneious v7 software. Contiguous sequences were aligned with the HIV-2 CAM-2 reference sequence (D00835) and a consensus sequence generated. All reads were mapped against the consensus to establish read depth and minority species.

The sequence of the 1st WHO IS for HIV-2 RNA described here is 9,496 bases long with a G+C content of 45.0%, representing the complete coding sequence with nine open reading frames (*gag, pol, vif, vpr, tat, rev, vpx, env*, and *nef*), as well as the U5 region of the 5′-long terminal repeat (5′-LTR) and U3 and R regions of the 3′-LTR of the HIV-2 genome. BLAST analysis (4) of this RNA template-derived sequence further confirmed the highest similarity with the HIV-2 subtype A CAM-2 proviral clone sequence (3) (total score 18,143, 99% identity, and 100% coverage). The mean read depth was 268,331-fold (±328,393 standard deviation [SD]) with a minimum of 16,285-fold. There are 140 positions with a minority nucleotide differing from the reference sequence with a frequency >3% and a base call accuracy of 99.9% (>Phred 30).

This is the first report of the complete genome sequence of the WHO HIV-2 RNA IS. The standard is used in development and evaluation of genome amplification assays for HIV-2 RNA quantification, which provide important clinical data for management of HIV-2-infected patients. The complete genome sequence reported here, derived from a viral RNA template, will support genome amplification assay development used in the clinical management of HIV-2-infected individuals and molecular epidemiological studies.

### Nucleotide sequence accession number.

The complete genome sequence of the WHO IS for HIV-2 RNA reported here has been deposited in GenBank under the accession number KU179861.
